# Development and validation of postoperative circulating tumor DNA combined with clinicopathological risk factors for recurrence prediction in patients with stage I-III colorectal cancer

**DOI:** 10.1186/s12967-023-03884-3

**Published:** 2023-01-30

**Authors:** Zhaoya Gao, Dandan Huang, Hui Chen, Yong Yang, Ke An, Changmin Ding, Zheping Yuan, Zhichao Zhai, Pengfei Niu, Qingkun Gao, Jinping Cai, Qingmin Zeng, Yanzhao Wang, Yuming Hong, Wanshui Rong, Wensheng Huang, Fuming Lei, Xiaodong Wang, Shiqing Chen, Xiaochen Zhao, Yuezong Bai, Jin Gu

**Affiliations:** 1 Department of Gastrointestinal Surgery, Peking University Shougang Hospital, No.9 Jinyuanzhuang Road, Shijingshan District, Beijing, China; 2 Department of Oncology, Peking University Shougang Hospital, Beijing, China; 3 Center for Precision Diagnosis and Treatment of Colorectal Cancer and Inflammatory Disease, Peking University Health Science Center, Beijing, China; 4 Medical Affairs, 3D Medicines, Inc., Shanghai, China; 5 Peking-Tsinghua Center for Life Sciences, Peking University, Beijing, China; 6 Key Laboratory of Carcinogenesis and Translational Research (Ministry of Education/Beijing), Department of Gastrointestinal Surgery, Peking University Cancer Hospital & Institute, Beijing, China; 7 Peking University International Cancer Institute, Beijing, China

**Keywords:** Colorectal cancer, Circulating tumor DNA, Clinicopathological risk factors, Recurrence

## Abstract

**Background:**

Circulating tumor DNA (ctDNA) detection following curative-intent surgery could directly reflect the presence of minimal residual disease, the ultimate cause of clinical recurrence. However, ctDNA is not postoperatively detected in ≥ 50% of patients with stage I-III colorectal cancer (CRC) who ultimately recur. Herein we sought to improve recurrence risk prediction by combining ctDNA with clinicopathological risk factors in stage I-III CRC.

**Methods:**

Two independent cohorts, both consisting of early-stage CRC patients who underwent curative surgery, were included: (i) the discovery cohort (N = 124) with tumor tissues and postoperative plasmas for ctDNA determination; and (ii) the external validation cohort (N = 125) with available ctDNA results. In the discovery cohort, somatic variations in tumor tissues and plasmas were determined via a 733-gene and 127-gene next-generation sequencing panel, respectively.

**Results:**

In the discovery cohort, 17 of 108 (15.7%) patients had detectable ctDNA. ctDNA-positive patients had a significantly high recurrence rate (76.5% vs. 16.5%, P < 0.001) and short recurrence-free survival (RFS; P < 0.001) versus ctDNA-negative patients. In addition to ctDNA status, the univariate Cox model identified pathologic stage, lymphovascular invasion, nerve invasion, and preoperative carcinoembryonic antigen level associated with RFS. We combined the ctDNA and clinicopathological risk factors (CTCP) to construct a model for recurrence prediction. A significantly higher recurrence rate (64.7% vs. 8.1%, P < 0.001) and worse RFS (P < 0.001) were seen in the high-risk patients classified by the CTCP model versus those in the low-risk patients. Receiver operating characteristic analysis demonstrated that the CTCP model outperformed ctDNA alone at recurrence prediction, which increased the sensitivity of 2 year RFS from 49.6% by ctDNA alone to 87.5%. Harrell's concordance index, calibration curve, and decision curve analysis also suggested that the CTCP model had good discrimination, consistency, and clinical utility. These results were reproduced in the validation cohort.

**Conclusion:**

Combining postoperative ctDNA and clinical risk may better predict recurrence than ctDNA alone for developing a personalized postoperative management strategy for CRC.

**Supplementary Information:**

The online version contains supplementary material available at 10.1186/s12967-023-03884-3.

## Background

Colorectal cancer (CRC) is currently the third most prevalent cancer worldwide, with approximately 1.9 million new cases diagnosed annually [1]. With the advancement of diagnostic techniques, a growing number of CRC patients are being diagnosed at an early stage before metastasis, for whom surgery is the preferred treatment method [2, 3]. After curative-intent surgery, the risk of cancer recurrence is estimated via a careful histological examination of the resected specimen, which subsequently determines whether patients should receive adjuvant chemotherapy (ACT). However, even after definitive therapy, 30–50% of patients still experience recurrence, with a significant percentage being in the low-risk population as identified by clinicopathological features [4, 5]. This demonstrates that the clinicopathology-based risk stratification is somewhat imprecise.

Toward this, a high volume of work has been conducted to improve the risk stratification of CRC patients to achieve better management. So far, efforts to refine recurrence risk for nonmetastatic CRC have mainly focused on examinations of the resected tumor to identify potential biomarkers with prognostic significance. Although multiple tissue-based biomarkers have been linked to recurrence risk, their hazard ratios are modest, typically ranging from 1.4 to 3.7, and the clinical application has remained debatable [4–7].

Circulating tumor DNA (ctDNA) analysis, also known as “liquid biopsy,” is an emerging and promising alternative strategy to directly evaluate the existence of minimal residual disease (MRD), the primary source of cancer recurrence. Several observational studies involving patients with solid tumors have shown that postoperative ctDNA is an important biomarker for predicting recurrence, redefining patient risk outcome groups, and guiding postoperative management [8–10]. Across various nonmetastatic CRC cohorts, patients with detectable ctDNA after curative-intent therapy have a very high probability of recurrence, typically no less than 80%, indicating high specificity of ctDNA for predicting disease recurrence [11–17]. But ctDNA is not found postoperatively in more than half of CRC patients who ultimately relapse, indicating its modest sensitivity [11, 15, 17]. Recently, the first interventional clinical trial with blood-based ctDNA assays in resected CRC was completed, and subgroup analysis showed that clinicopathological risk factors could still differentiate the risk of relapse in ctDNA-negative patients [18]. According to this research, combining ctDNA and clinicopathological factors might allow for better risk stratification and postoperative decision-making. However, no study has built an effective model utilizing ctDNA and clinicopathological factors to predict recurrence in CRC patients undergoing radical surgery.

Herein, we adopted a tumor-informed assay with a fixed panel to determine ctDNA and evaluate the clinical validity of postoperative ctDNA for recurrence prediction. Then we developed a comprehensive model utilizing ctDNA and clinicopathological risk factors (CTCP) to predict recurrence after radical resection in CRC patients and validated the CTCP model in an independent cohort. These findings will shed some light on postoperative management strategies.

## Methods

### Study design and population

There were three major phases of the study: (1) determine postoperative ctDNA status and evaluate its prognostic value in the discovery cohort; (2) construct the CTCP model for predicting the risk of CRC recurrence after radical surgery in the discovery cohort; and (3) validate the CTCP model’s performance in an external validation cohort.

**Fig. 1:**
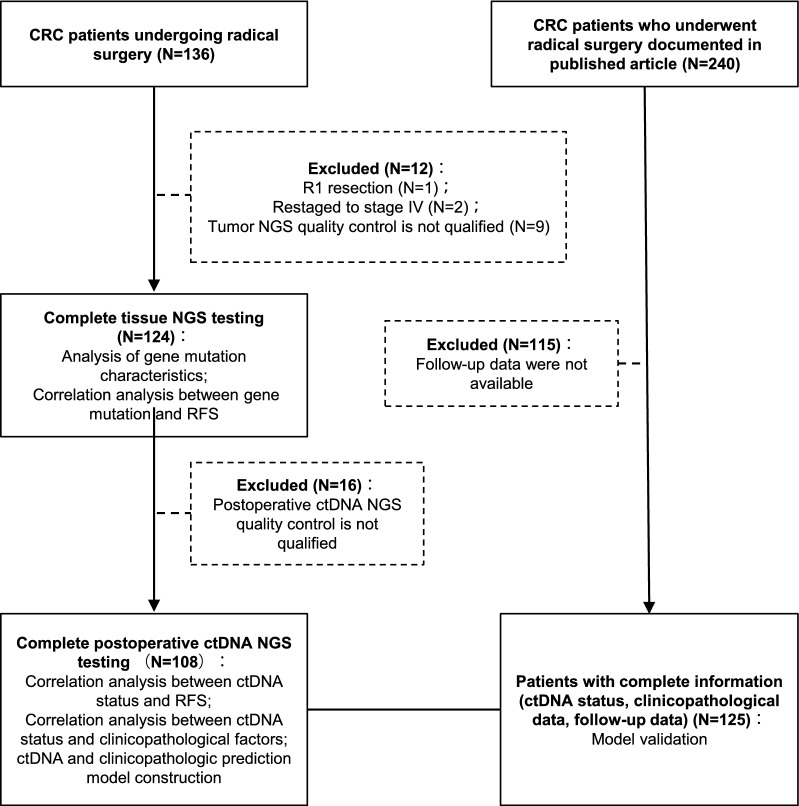
Flowchart depicting the patient selection, sample collection, and study overview. *CRC* colorectal cancer, *NGS* next-generation sequencing, *RFS* recurrence-free survival, *ctDNA* circulating tumor DNA

We collected data on patients with stage I-III CRC who underwent curative-intent surgery without neoadjuvant chemotherapy at Peking University Shougang Hospital between February 2017 and July 2020 to establish the discovery cohort. Figure 1 shows the criteria for establishing the discovery cohort. Tumor tissue was obtained at surgery, and blood was drawn on postoperative days 7–10. ctDNA analyses were performed retrospectively by 3D Medicines Inc. (Shanghai, China). Microsatellite instability (MSI) status was assessed simultaneously via the technique previously published [19]. The use of ACT after surgery was at the discretion of the treating clinicians, who were blinded to the ctDNA results. Medical records were reviewed for clinicopathologic variables, including age at diagnosis, sex, primary tumor location, tumor differentiation, histological type, pathological grade, lymph node yield, lymphovascular invasion, nerve invasion, and preoperative carcinoembryonic antigen (CEA) level.

The validation dataset was derived from a published observational study designed to assess whether postoperative serial ctDNA measurements predict high recurrence risk in patients with stage II/III CRC and identify recurrence earlier than conventional imaging [13]. This trial recruited 276 patients with stage II/III CRC who were treated with curative intent. We downloaded the data of these patients. Of 276 patients, 125 had available ctDNA results from the plasma collected on postoperative days 3–7, clinicopathologic features, and follow-up data, which composed a validation set. As for clinicopathologic variables, in the discovery cohort, histological type of 7 patients and preoperative CEA level of 4 patients were missing; in the validation cohort, preoperative CEA level of 4 patients was missing. These missing data were imputed using multiple imputations.

Recurrence-free survival (RFS) was defined from the date of surgery to disease recurrence or death, whichever happened first. The Ethics Committee of Peking University Shougang Hospital approved the protocols (Approval ID: IRBK-2020-045-01), which complied with the principles of the Declaration of Helsinki.

### Tumor tissue mutational analysis and ctDNA detection

Tumor tissues from surgical resection were analyzed for somatic mutations by targeted next-generation sequencing (NGS) of 733 cancer-related genes as previously reported [20, 21]. Germline mutations identified by the 733-gene NGS panel on matched peripheral blood leukocytes were filtered out before formal analysis of tumor sequencing data. Median de-duped sequencing depths for tumor tissue and peripheral blood leukocytes were 538 × and 312 × , respectively. All somatic variants identified in the primary tumor of every patient were labeled as patient-specific somatic variants for further ctDNA tracking. Plasma samples were detected using a 127-gene NGS panel, and this panel was customized for MRD testing after radical resection of CRC, achieving a median de-duped sequencing depth of 11,014 × according to methods published previously [22–24]. To improve specificity, especially for variants with low allele frequency in the ctDNA, an in-house loci specific variant detection algorithm called MTI (Maximized Tumor-Informed) was applied. Briefly, a combined model of binomial test and in-silico noise reduction for each tumor-informed site was used to remove false-positive variants. Patients with one or more patient-specific somatic variants in plasmas were considered as ctDNA-positive, and ctDNA-negative otherwise.

### Construction and performance evaluation of the CTCP model for predicting the risk of recurrence

The ctDNA status and clinicopathological factors in the discovery cohort, including age, sex, primary tumor location, MSI status, histological type, pathologic stage, lymphovascular invasion, nerve invasion, and preoperative CEA level, were submitted to univariate Cox regression analyses. Variables reaching P < 0.10 on univariate analysis were combined to construct a CTCP model using cph function from the R package “rms.” The weight of each variable in this model corresponded to the respective coefficients from the multivariable Cox regression analysis. The risk score was computed per the specific risk score formula. The optimal cut-off point was estimated by maximizing the Youden index on the receiver operating characteristic (ROC) curve. According to this threshold, all participants in the discovery cohort were separated into high-and low-risk groups. Kaplan-Meier survival curves combined with a log-rank test were used to examine the survival differences between the high-and low-risk groups. The predictive accuracy of the CTCP model for RFS was assessed by the area under the ROC curve (AUC). The CTCP model’s discrimination, calibration, and clinical application for RFS prediction were also evaluated, wherein the discrimination was measured using Harrell's concordance index (c-index), the calibration by comparing predicted and observed RFS, and the clinical application via decision curve analysis (DCA). Additionally, the CTCP model's performance was assessed in an external validation cohort from the Sun Yat‑Sen University Cancer Center (Guangzhou, China) [13].

### Statistical analyses

Clinicopathological variables were summarized as frequencies/percentages for categorical variables and median (range) for continuous covariates. Categorical variables were compared using Chi-square or Fisher's exact tests as appropriate, and continuous variables were compared using Mann-Whitney U test. RFS was calculated using the Kaplan-Meier method, and differences between groups were analyzed using the log-rank test. Univariable and multivariable Cox regression analyses were performed to construct the prediction model and calculate the independent risk factors. The R package “DynNom” was used to develop the web-based nomogram. The missing data of patient characteristics were imputed using the R package “mice.” To evaluate this model, the AUC, comparison between AUCs, calibration curves, and DCA were computed with the R package "surivivalROC," “survcomp,” “rms,” and “dcurves,” respectively. All analyses were completed in R version 4.1.2 (https://www.r-project.org/). P values below 0.05 were considered statistically significant, unless otherwise noted.

## Results

### Clinicopathological characteristics of the discovery cohort

Patient selection, sample collection, and study overview are presented in Fig. 1. We reviewed 136 patients with stage I-III CRC who underwent curative-intent surgery without neoadjuvant chemotherapy. Twelve patients were excluded as they underwent an R1 resection (N = 1), were restaged to stage IV (N = 2), or had unqualified tumor samples for library preparation (N = 9), leaving 124 patients for analysis. Table 1 presents a summary of patient clinicopathological characteristics. The median age was 61 years (range 29–75), 65.3% (81/124) were male, 24.2% (30/124) had right-sided tumors, and 38.3% (46/124) exhibited abnormal preoperative CEA levels (> 5 ng/ml). Based on pathological characteristics, 15.3% (19/124) of cases were diagnosed as stage I, 41.9% (52/124) as stage II, and 42.7% (53/124) as stage III, 7.7% (9/124) had poorly differentiated tumors, 19.4% (24/124) had lymphovascular invasion, 14.5% (18/124) had nerve invasion, and 8.9% (11/124) had MSI-high tumors. After surgery, 67 (54.0%) patients, including 23 stage II and 44 stage III, received ACT at the discretion of their clinicians. During a median follow-up of 36.7 months (range, 8.1–55.1 months), 32 (25.8%) patients experienced recurrence, including one stage I, five stage II, and 26 stage III.

**Table 1:** Basic characteristics of the patients with stage I-III CRC in the discovery cohort

Clinicopathological parameters	All patients (N = 124)
Sex	
Male	81 (65.3%)
Female	43 (34.7%)
Age	
Median [range]	61 (29–75)
Stage	
I	19 (15.3%)
II	52 (41.9%)
III	53 (42.7%)
Tumor location	
Left	94 (75.8%)
Right	30 (24.2%)
Histologic type	
Adenocarcinoma	114 (91.9%)
Mucinous	10 (8.1%)
Histopathological differentiation grade	
Poor	9 (7.7%)
Medium/Well	108 (92.3%)
Lymphatic/vascular invasion	
No	100 (80.6%)
Yes	24 (19.4%)
Nerve invasion	
No	106 (85.5%)
Yes	18 (14.5%)
MSI status	
MSS	113 (91.1%)
MSI-H	11 (8.9%)
pre_CEA (ug/L)	
< = 5	74 (61.7%)
> 5	46 (38.3%)
Adjuvant chemotherapy	
No	57 (46.0%)
Yes	67 (54.0%)

*MSS* microsatellite stable, *MSI-H* microsatellite instability-high

In the primary tumor, somatic variants were found in 123 of 124 (99.2%) patients with a median number of 8 (range, 1–172) per case. Additional file 1: Figure S1 shows the detailed mutational landscape along with corresponding clinicopathological characteristics. Univariate Cox regression for RFS revealed no gene variants significantly associated with RFS.

### ctDNA status and its association with recurrence risk

**Fig. 2:**
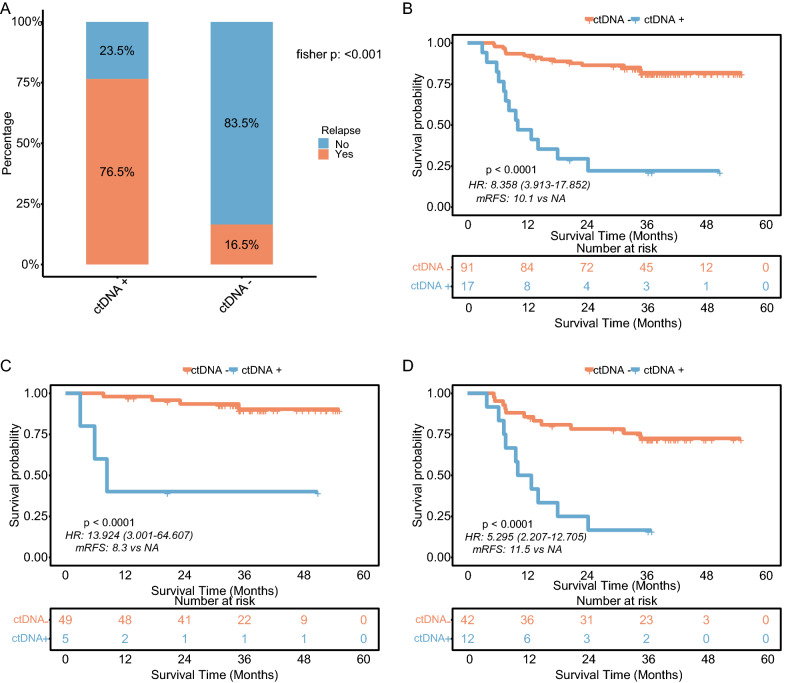
RFS outcomes according to ctDNA status in the discovery cohort. **A** The recurrence rate for patients stratified by postoperative ctDNA status. **B** Kaplan-Meier curves of RFS for patients stratified by postoperative ctDNA status. **C** and **D** Kaplan-Meier curves of RFS for patients stratified by postoperative ctDNA status in the subset of patients with or without ACT administration. *CRC* colorectal cancer, *RFS* recurrence-free survival, *ctDNA* circulating tumor DNA, *ACT* adjuvant chemotherapy

Postoperative plasma samples of 108 patients passed NGS quality control, wherein 17 (15.7%) were classified as ctDNA-positive and 91 as ctDNA-negative. The relationship analysis between ctDNA status and clinicopathological parameters revealed that a positive ctDNA finding was significantly associated with nerve invasion and tumor stage (Additional file 1: Table S1). Among these 17 ctDNA-positive patients, 2 were stage II, and 15 were stage III. The recurrence rate of 76.5% (13/17) for the ctDNA-positive patients was significantly higher than that of 16.5% (15/91) for the ctDNA-negative patients (P < 0.001; Fig. 2A). The Kaplan–Meier survival curves presented that ctDNA-positive patients had a significantly poor RFS versus the ctDNA-negative patients (median RFS 10.1 months vs. not reached [NR], HR = 8.36; 95% confidence interval [CI], 3.91–17.85; P < 0.001; Fig. 2B). Subgroup analysis demonstrated that the poor RFS for ctDNA-positive patients applied to different stages and receiving/not receiving ACT, indicating that the relationship between ctDNA status and RFS was independent of tumor stage and ACT (Fig. 2C–D, Additional file 1: Figure S2A-B). Together, these data showed the potentiality of ctDNA as a biomarker for predicting RFS. The performance of ctDNA for predicting RFS at 6–48 months is shown in Additional file 1: Table S2, with a sensitivity of 44.7–60% and a specificity of 86.4–95.9%. At the time point of 24 months, ctDNA status presented a 49.6% sensitivity and 86.5% specificity for predicting recurrence.

### CTCP-score model construction and its performance evaluation

**Fig. 3:**
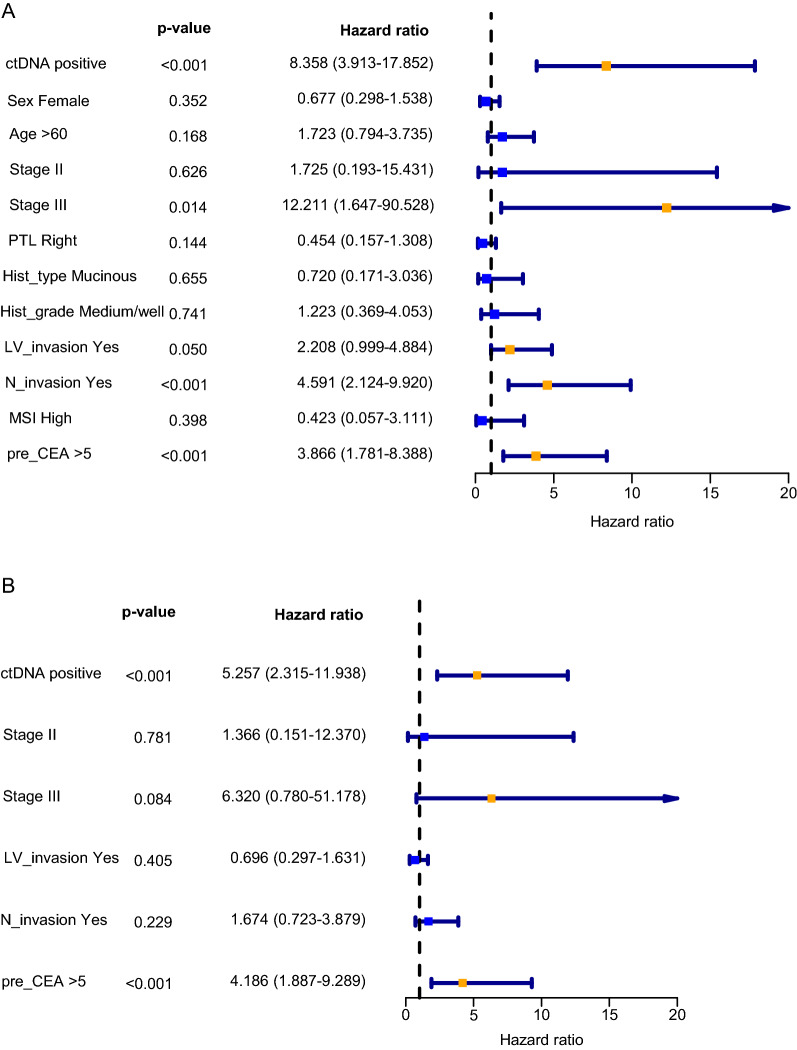
Univariate and multivariate Cox analysis of RFS by clinicopathological variables and postoperative ctDNA status. **A** Univariate Cox regression is used to screen factors potentially correlated with RFS (P < 0.1). **B** The factors with P < 0.1 in the univariate Cox regression are included in the multivariate Cox regression to calculate the independent risk factors. *RFS* recurrence-free survival, *ctDNA* circulating tumor DNA, *PTL* primary tumor location, *LV_invasion* lymphovascular invasion, *N_invasion* nerve invasion, *MSI* microsatellite instability, *pre_CEA* preoperative carcinoembryonic antigen

The data above showed that ctDNA has a high specificity but a modest sensitivity, consistent with previous reports [11, 15–17]. Here, we tried to better stratify the risk of recurrence by combining clinicopathological factors with ctDNA status in the discovery cohort. Univariate regression analysis was used to analyze the ctDNA status and traditional clinicopathological factors that might affect the recurrence. Factors with P < 0.1, including ctDNA status, tumor stage, lymphovascular invasion, nerve invasion, and preoperative CEA level, were further included in the multivariate analysis (Fig. 3A–B). The weight of each parameter was determined by regression coefficients in the multivariable Cox regression analysis to construct the CTCP model. The risk score for each patient was calculated using the following formula generated by the CTCP model: CTCP score = Exp (1.6595879 × ctDNA) + (0.31218627 × Stage II) + (1.84369027 × Stage III) + (− 0.3620769 × Lymphovascular invasion) + (0.51542913 × Nerve invasion) + (1.43177661 × Preoperative CEA). ctDNA status was the most significant and independent prognostic factor associated with recurrence risk (HR = 6.0; 95% CI 2.2 to 16, P < 0.001; Fig. 3B).

**Fig. 4:**
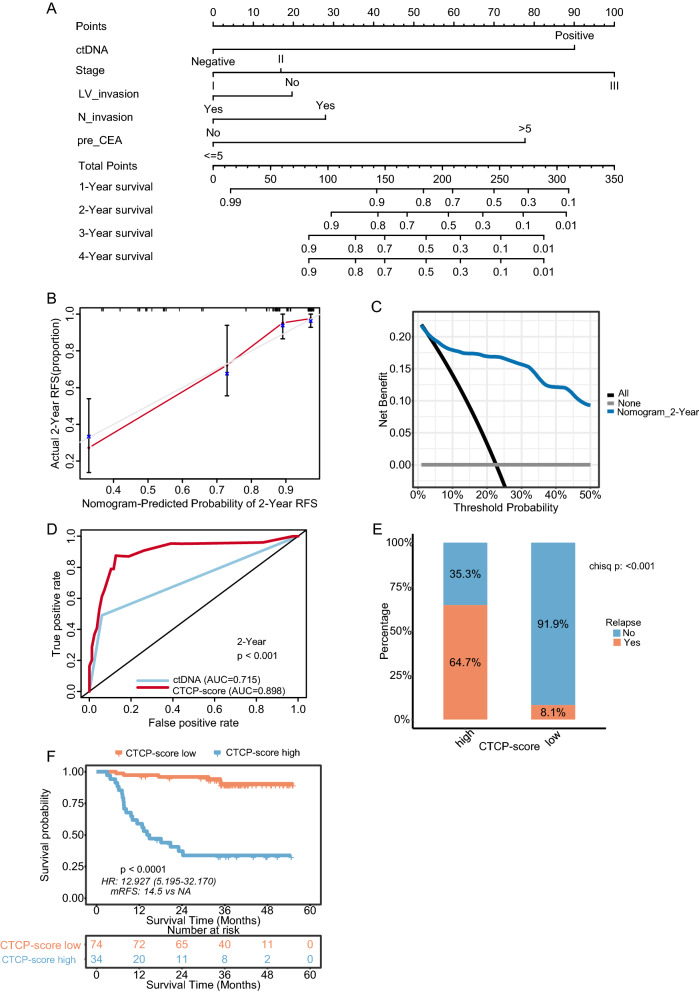
The performance evaluation of the CTCP model for predicting RFS in the discovery cohort. **A** The predictive model is visualized into a five-factor nomogram to predict RFS in patients with stage I-III CRC after curative surgery. **B** The calibration curves show the concordance between model prediction and actual distribution for 2 year RFS. Gray line: reference line. Red line: the prediction curve generated by this model. **C** The DCA curves show the net clinical benefit of the application of the CTCP model to predict 2 year RFS. **D** ROC analysis is used to compare the CTCP model with the ctDNA alone to predict 2 year RFS. **E** The recurrence rate for CRC patients stratified by the CTCP model. **F** Kaplan–Meier curves of RFS for CRC patients stratified by the CTCP model. *CTCP* ctDNA and clinicopathological risk factors, *CRC* colorectal cancer, *RFS* recurrence-free survival, *ctDNA* circulating tumor DNA, *ROC* receiver operating characteristic, *DCA* decision curve analysis

To facilitate the clinical application of this score, we established a nomogram to visualize this model. The point scale assigned a score to each subtype of these variables. RFS probability could be calculated at the time points of 1-, 2-, 3-, and 4 year by summing the scores of each variable and placing the sum on the survival rate scale (Fig. 4A). The nomogram has been deployed as a freely accessible online calculator at https://oncologyusage.shinyapps.io/dynnom_crc/. The c-index value of the CTCP model was 0.855 suggesting a significant prognosis value of discrimination. The calibration plots, which ran very close to the diagonal, presented an excellent agreement between the CTCP prediction and actual observation for 1-, 2-, 3-, and 4 year RFS (Fig. 4B, Additional file 1: Figure S3A–C). The DCA indicated that the application of the CTCP model to predict RFS yielded more net benefit across almost the entire range of risk thresholds than the “treat all” or “treat none” approach (Fig. 4C, Additional file 1: Figure S3D–F).

Each patient was assigned a risk score per the risk score formula. With the optimal cut-off value of 10.59095 in the discovery cohort, 34 patients were assigned to the high-risk group, and 74 patients were assigned to the low-risk group. The high-risk group had a significantly higher recurrence rate (64.7% vs. 8.1%, P < 0.001) and shorter RFS (median RFS 14.5 months vs. NR, HR = 12.93, 95% CI 5.20–32.17, P < 0.001) versus the low-risk group (Fig. 4E–F). The 2 year and 3 year RFS for the high-risk group were 37.2% and 33.8%, respectively, and 95.9% and 90.4% for the low-risk group.

Notably, the prognosis gap between the high- and low-risk groups looked much more impressive than that between ctDNA-positive and -negative patients (HR = 12.93 vs. 8.36). Additionally, the AUC values of the CTCP model were significantly or nearly-significantly higher than those of ctDNA alone for predicting 1-, 2-, 3- and 4 year RFS (1 year AUC: 0.910 vs. 0.738, P = 0.176; 2 year AUC: 0.898 vs. 0.715, P < 0.001; 3 year AUC: 0.883 vs. 0.700, P < 0.001; and 4 year AUC: 0.883 vs. 0.700, P < 0.001) (Fig. 4D, Additional file 1: Figure S3G–I). The performance of the CTCP model for predicting RFS at different time points is shown in Additional file 1: Table S3. Compared with ctDNA alone, the CTCP model markedly increased the sensitivity for predicting recurrence with a small negative effect on the specificity. For example, sensitivity for 2 year RFS prediction increased from 49.6% to 87.5%, and specificity changed from 94.7 to 88.2%.

### Validation of the CTCP model

**Fig. 5:**
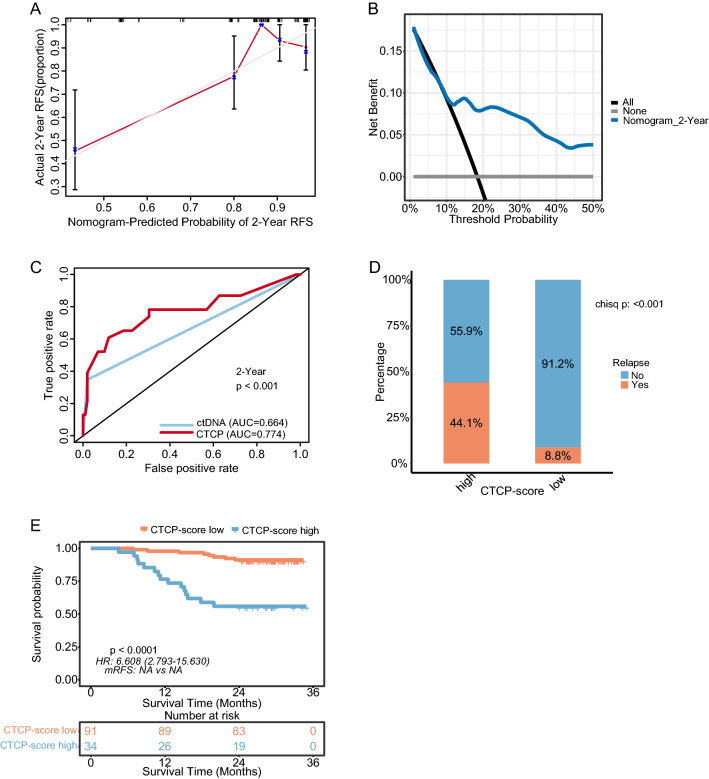
The performance evaluation of the CTCP model for predicting RFS in the validation cohort. **A** The Calibration curves reveal the concordance between model prediction and actual distribution for 2 year RFS. Gray line: reference line. Red line: the prediction curve generated by this model. **B** The DCA curves show the net clinical benefit of the application of the CTCP model to predict 2 year RFS. **C** Comparison of the CTCP model with ctDNA using ROC analyses for predicting 2 year RFS. **D** The recurrence rate for CRC patients stratified by the CTCP model. **E** Kaplan–Meier curves of RFS for CRC patients stratified by the CTCP model. *CTCP* ctDNA and clinicopathological risk factors, *CRC* colorectal cancer, *RFS* recurrence-free survival, *ctDNA* circulating tumor DNA, *ROC* receiver operating characteristic, *DCA* decision curve analysis

The results were replicated in another independent cohort (N = 125) to confirm the CTCP model’s predictive ability. In this cohort, we observed similar results. The calibration curves of this model exhibited good fitness (Fig. 5A, Additional file 1: Figure S4A). DCA curves demonstrated that the CTCP model provided a net benefit over the “treat-all” or “treat-none” strategy across a wide range of threshold probability (Fig. 5B, Additional file 1: Figure S4B). The AUC values of this model were significantly higher than those of ctDNA alone for predicting 1- and 2 year RFS (1 year AUC: 0.900 vs. 0.783, P = 0.029; and 2 year AUC: 0.774 vs. 0.664, P < 0.001) (Fig. 5C, Additional file 1: Figure S4C). Likewise, the risk score of each patient was determined per the risk-score formula. Based on this cohort's optimal cut-off value of 10.59095, the patients were split into the high-risk group (N = 34) and the low-risk group (N = 91). A significantly higher recurrence rate (53.8% vs. 9.1%, P < 0.001) and shorter RFS (median RFS 18.9 months vs. NR, HR = 8.38, 95% CI 3.61–19.47, P < 0.001) were observed in the high-risk group versus the low-risk group (Fig. 5D–E). Of note, the optimal cut-off values in the discovery and validation cohorts are identical, suggesting the strong robustness of our model.

## Discussion

There is an urgent need for a valid and sensitive marker to predict the prognosis of CRC patients following radical resection to optimize individually tailored postoperative management strategies. Here, we evaluated the prognostic role of postoperative ctDNA in patients with stage I-III CRC and revealed ctDNA status as the most significant and independent predictor of RFS. The sensitivity and specificity of 2 year RFS predicted by ctDNA alone were 49.6% and 94.7%, respectively. We combined ctDNA with clinicopathological risk factors to construct the CTCP prediction model, which showed a better predictive value than ctDNA alone for RFS in patients with stage I-III CRC and increased the sensitivity for 2 year RFS to 87.5%. Its predictive value was also validated externally. To simplify the CTCP model’s use, we have made it available as a free web-based calculator. To our knowledge, this is the first comprehensive study to integrate clinical risk with ctDNA to examine the prognostic value for guiding postoperative management strategies in stage I-III CRC.

Currently, two ctDNA analysis strategies for MRD detection are available: a tumor-informed (fixed or personalized) and a tumor-agnostic approach (also referred to as tumor-uninformed or plasma-only ctDNA) [25]. As the name implies, the former approach was based on tumor sequencing and could precisely weed out non-tumor-derived alterations. For the tumor-agnostic strategy, ctDNA analysis does not rely on a priori of tumor profiling and often includes broad panel-based sequencing. Although the tumor-informed approach was more resource-intensive, it offers higher analytical sensitivity and specificity and is especially suitable for the detection of MRD. In this study, we adopted the fixed tumor-informed assay with a custom CRC panel, achieving a median de-duped sequencing depth of 11014 × , higher than the fixed tumor-informed assays previously reported [13, 26]. This high-depth sequencing enables the ctDNA with a very low variant allele frequency (VAF) to be detected, guaranteeing MRD detection sensitivity. Additionally, we used a novel loci specific variant detection algorithm in this study to remove false-positive variants to improve specificity, especially for variants with low allele frequency in the ctDNA. There were three patients with gene variations at VAF ≤ 0.05% in the 17 ctDNA-positive patients, wherein one carried APC p.D1486lfs*21 with a VAF of 0.02%, one harbored APC p.T493Rfs*20 with a VAF of 0.02%, and one had SMAD p.R361C with a VAF of 0.05%, and they all experienced relapse between 10 and 18 months after the operation.

We observed a significant association between ctDNA-positive status and pathological tumor stage. The percentage of ctDNA-positive patients increased with the progression of pathology stage, with 0% for stage I, 4.5% for stage II, and 32.6% for stage III, generally consistent with previous reports which measured ctDNA using Safe-Sequencing System and Signatera, two representative tumor-informed personalized technologies [11, 17]. In these two technologies, one or several patient-specific somatic variants are targeted for detection, which allows the employment of ultra-deep sequencing to achieve high sensitivity and specificity [27]. Encouragingly, the sensitivity and specificity for RFS prediction of postoperative ctDNA detected by our technology were comparable to those of postoperative ctDNA determined by Safe-Sequencing or Signatera ctDNA assays (the sensitivity of 44.7%–60% and the specificity of 86.4%–95.9% by our strategy vs. sensitivity of about 40% ~ 50% and the specificity of 86% ~ 97% by Safe-Sequencing and Signatera ctDNA assays) [11, 12, 14, 16]. Accordingly, the similar detection rate of ctDNA and sensitivity of ctDNA to predict recurrence generated comparable hazard ratios between postoperative ctDNA-positive and -negative patients by our ctDNA detection technique, Safe-Sequencing System and Signatera ctDNA assays (HR of 8.358 by our strategy vs. HR of 3.8 ~ 18 by Safe-Sequencing and Signatera ctDNA assays) [11, 12, 14, 16]. Additionally, compared to the personalized tumor-informed approach Safe-Sequencing System and Signatera ctDNA assays, our fixed tumor-informed ctDNA detecting strategy could offer several advantages, such as rapid turnaround time, potential cost savings, decreased logistical complexity, and the possibility to use in a large set of laboratories around the world. Together, these findings demonstrated the validity of our fixed NGS panel-based ctDNA detection strategy and the feasibility of ctDNA status determined by our ctDNA detection method for recurrence prediction and risk stratification.

Of note, the prognosis gap between ctDNA-positive and -negative patients in the non-ACT subgroup was more prominent than in the ACT subgroup (HR = 12.93 vs. 8.358), suggesting that ACT administration may decrease the prognostic impact of ctDNA by increasing MRD clearance. In our study, there were four ctDNA-positive patients without documented recurrence, of which one was on follow-up for less than 2 years, and two received ACT (Additional file 1: Figure S2C). These findings tended to favor that ctDNA-positive patients might benefit more from adjuvant regimens [28]. Randomized clinical trials are required to investigate to what extent ctDNA-positive and ctDNA-negative patients may benefit from standard ACT.

Our technology demonstrated satisfactory sensitivity and specificity in comparison with previous studies. However, even though our technology achieved the limit of detection with 0.01% VAF, about half of the patients who experienced recurrences eventually had no detectable ctDNA after the operation. Current efforts to increase sensitivity for ctDNA detection have focused primarily on serial ctDNA monitoring. It is expected and proven that as time passes after surgery, progression of any MRD would result in a higher percentage of individuals with detectable ctDNA [12, 29]. Indeed, postoperative serial ctDNA detection does improve sensitivity [11, 13, 16, 18]. But there is no doubt that serial ctDNA analysis increases costs. In addition, for patients who are postoperatively ctDNA negative but eventually relapse, serial ctDNA measurements are still passively waiting for the progression of MRD until the release of ctDNA above the detection threshold. Thus, the ability to pinpoint which individuals would be more likely to relapse despite postsurgical ctDNA negative could potentially allow placing these individuals on an accelerated path to get additional therapy or intensive observation.

Given that clinicopathological factors reflect the intrinsic characteristics of tumors and are critical considerations for clinical decision-making [30–33], we combined ctDNA and clinical risk to construct the CTCP model and redefine the risk of recurrence. All clinicopathological risk factors included in this model were acquired during routine CRC treatment, which increased the cost-effectiveness. Compared with ctDNA alone, the CTCP model improved the sensitivity for predicting recurrence while retaining a relatively high specificity. Most notable was the 2 year RFS of 95.8% and 3 year RFS of 90.4% among patients with low-risk disease, indicating that the reduction of the intensity of surveillance might be considered for this low-risk population. Expectedly, this would translate into a substantial reduction in surveillance costs since most patients fall into this category. A larger number of prospective, multicenter studies are required to confirm our findings before they can be applied to daily clinical practice. Alongside this, the published data showed that serial ctDNA-negative patients after definitive therapy have a 2 year RFS of 96% in stage II-III CRC and a 3 year RFS of 92.5% in stage II colon Cancer [13, 18]. Here, by combining with clinical risk, a single detection of ctDNA yielded a comparable prognostic value with serial ctDNA measurements. Furthermore, our CTCP model also presented a similar prognostic value with the combined model of two time point ctDNA testing, consensus molecular subtype classification, and clinical risk recently reported by Li et al*.* [26], wherein the 2 year and 3 year RFS of the low-risk patients defined by the model were both 93.5%. In line with the opinions of Li and colleagues, our results also indicated that despite the significant prognostic value of postoperative ctDNA, the role of clinicopathological features of tumors could not be overlooked in individualized treatment and surveillance.

The primary strength of our study is that the CTCP model is externally validated and achieves good performance in the validation set. Unexpectedly, the discovery and validation cohorts’ optimal cut-off values align, highlighting our model’s robustness. There are potential limitations in this study. In addition to its retrospective nature, our conclusions are limited by the small number of postoperative ctDNA-positive patients despite our study’s relatively large number of patients. Notwithstanding this, our finding that postoperative ctDNA is a robust predictor of cancer recurrence is in line with a number of recent reports [11–17]. Additionally, postoperative serial ctDNA detection is not performed, and thus we could not directly compare the performance of CTCP and serial ctDNA measurements in predicting recurrence. Further randomized clinical trials are warranted to determine whether ACT and surveillance could be de-escalated in the low-risk patients defined by the CTCP model and escalated in the high-risk patients.

## Conclusions

We developed and validated the CTCP model that better predicts recurrence than ctDNA alone. This model provides a timely and cost-effective approach to identifying patients at high risk of recurrence that may hopefully be further verified in a prospective trial.

## Supplementary Information

**Additional file 1: Figure S1.** Heatmap demonstrating the mutational landscape of the primary tumors from 124 evaluable CRC patients. **Figure S2.** RFS outcomes of ctDNA-positive and ctDNA-negative CRC patients with different pathologic stages. **Figure S3. **Evaluation of the CTCP model for predicting 1-, 3- and 4 year RFS in the discovery cohort. **Figure S4.** Evaluation of the CTCP model for predicting 1 year RFS in the validation cohort. **Table S1.** The relationship analysis between postoperative ctDNA status and clinicopathological parameters. **Table S2.** The sensitivity and specificity of postoperative ctDNA status for predicting RFS at 6-48 months. **Table S3.** The sensitivity and specificity of the CTCP model for predicting RFS at 6-48 months.

## Data Availability

The datasets supporting the conclusions of this article are included within the article and its additional files.

## Abbreviations

ctDNA: Circulating tumor DNA
CRC: Colorectal cancer
CTCP: CtDNA and clinicopathological risk factors
ACT: Adjuvant chemotherapy
MRD: Minimal residual disease
MSI: Microsatellite instability
CEA: Carcinoembryonic antigen
RFS: Recurrence-free survival
NGS: Next-generation sequencing
ROC: Receiver operating characteristic
AUC: Area under the ROC curve
c-index: Concordance index
DCA: Decision curve analysis
VAF: Variant allele frequency
