# Supplementary material for: Development and validation of postoperative circulating tumor DNA combined with clinicopathological risk factors for recurrence prediction in patients with stage I-III colorectal cancer
Source: J Transl Med. 2023 Jan 30;21:63. doi: 10.1186/s12967-023-03884-3 (PMC9887832; doi:10.1186/s12967-023-03884-3)
Supplement: Supplementary file 1 — Additional file 1: Figure S1. Heatmap demonstrating the mutational landscape of the primary tumors from 124 evaluable CRC patients. Figure S2. RFS outcomes of ctDNA-positive and ctDNA-negative CRC patients with different pathologic stages. Figure S3. Evaluation of the CTCP model for predicting 1-, 3- and 4 year RFS in the discovery cohort. Figure S4. Evaluation of the CTCP model for predicting 1 year RFS in the validation cohort. Table S1. The relationship analysis between postoperative ctDNA status and clinicopathological parameters. Table S2. The sensitivity and specificity of postoperative ctDNA status for predicting RFS at 6-48 months. Table S3. The sensitivity and specificity of the CTCP model for predicting RFS at 6-48 months. [file 12967_2023_3884_MOESM1_ESM.docx]

***Online-Only Supplement***

***Supplement to: Gao, et al.*** ***Development and validation of postoperative circulating tumor DNA combined with clinicopathological risk factors for recurrence prediction in patients with stages I-III colorectal cancer***

**Figure S1** Heatmap demonstrating the mutational landscape of the primary tumors from 124 evaluable CRC patients


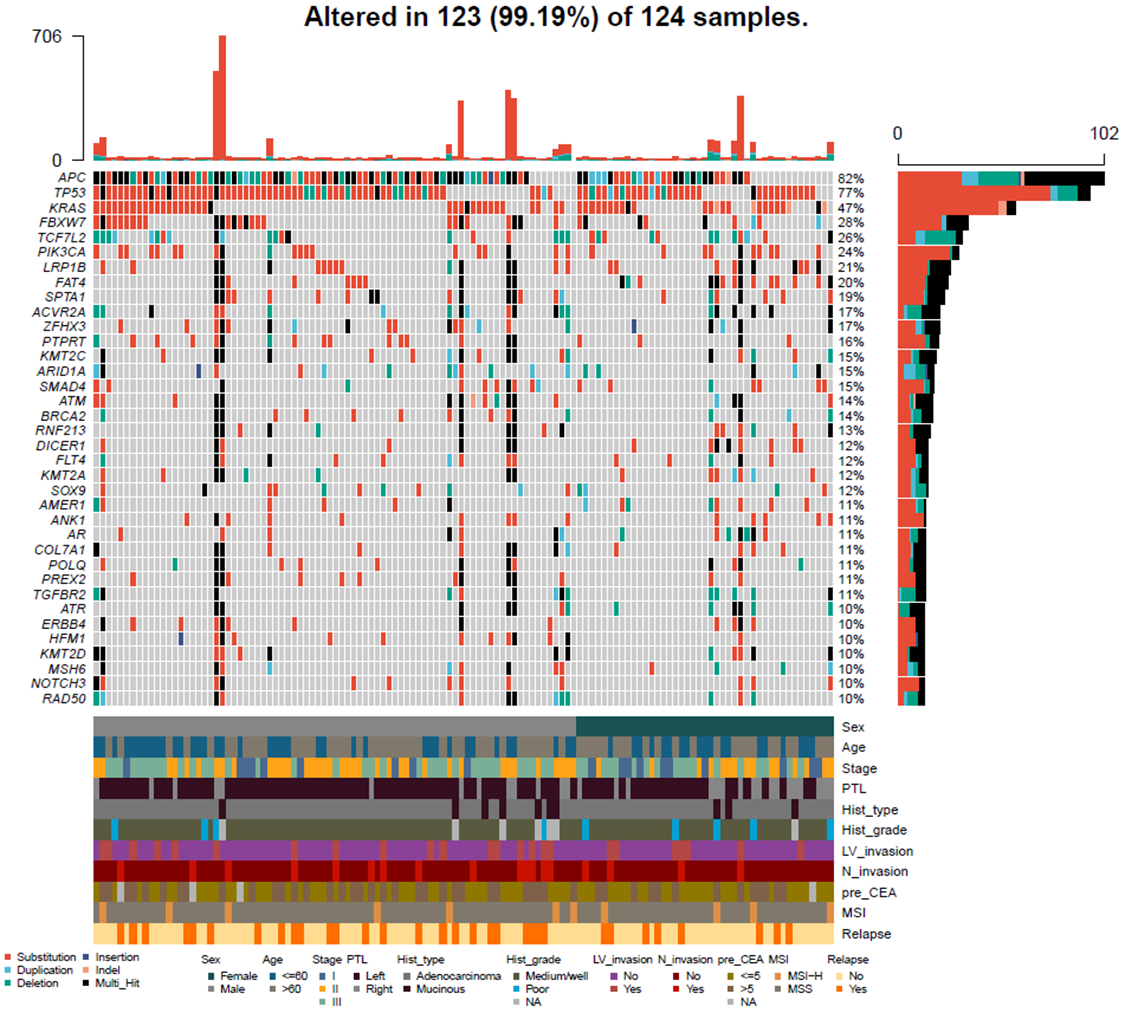


CRC, colorectal cancer; PTL: primary tumor location; LV_invasion: lymphovascular invasion; N_invasion: nerve invasion; MSI: microsatellite instability; pre_CEA, preoperative carcinoembryonic antigen.

**Figure S2** RFS outcomes of ctDNA-positive and ctDNA-negative CRC patients with different pathologic stages.


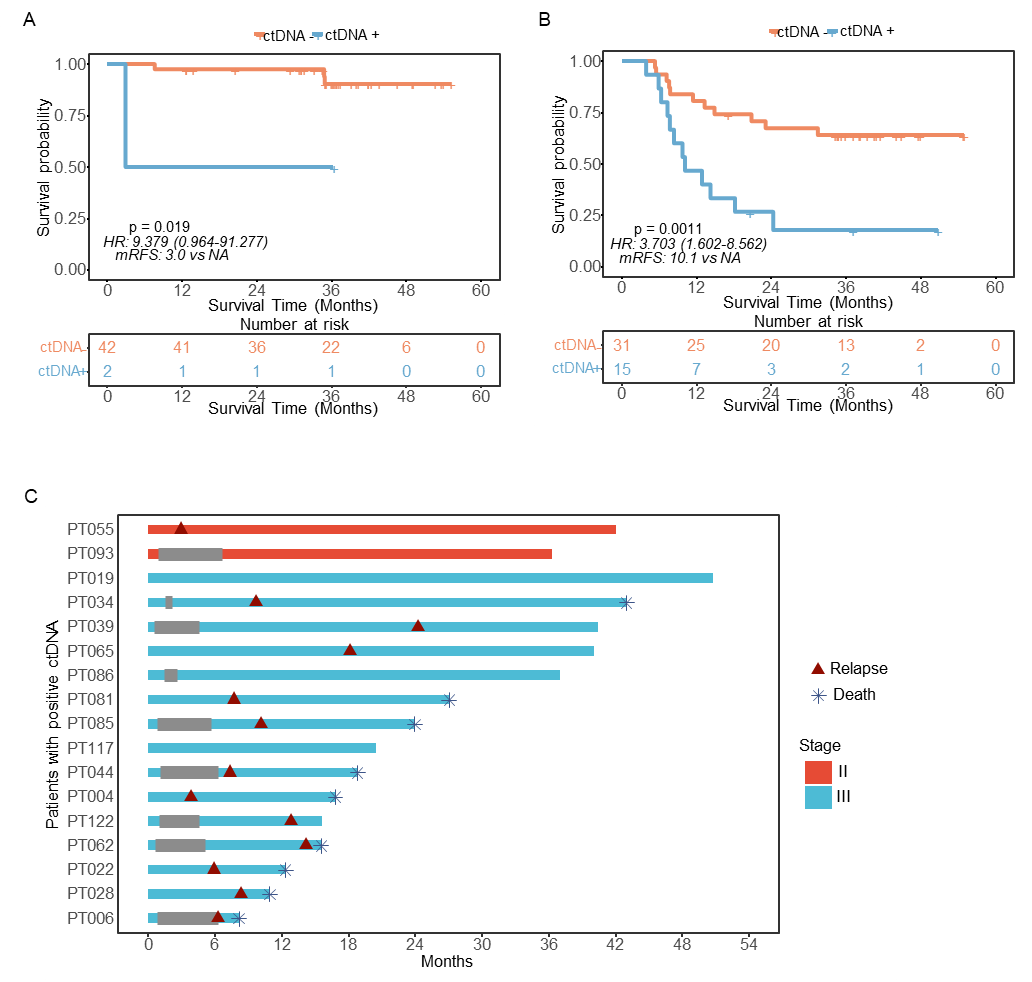


(A and B) Kaplan-Meier curves of RFS for patients stratified by postoperative ctDNA status in the subset of patients with pathologic stage II or III. (C) The clinical courses of 17 postoperative ctDNA‑positive patients.

CRC, colorectal cancer; RFS: recurrence-free survival; ctDNA: circulating tumor DNA.

**Figure S3** Evaluation of the CTCP model for predicting 1-, 3- and 4-year RFS in the discovery cohort.


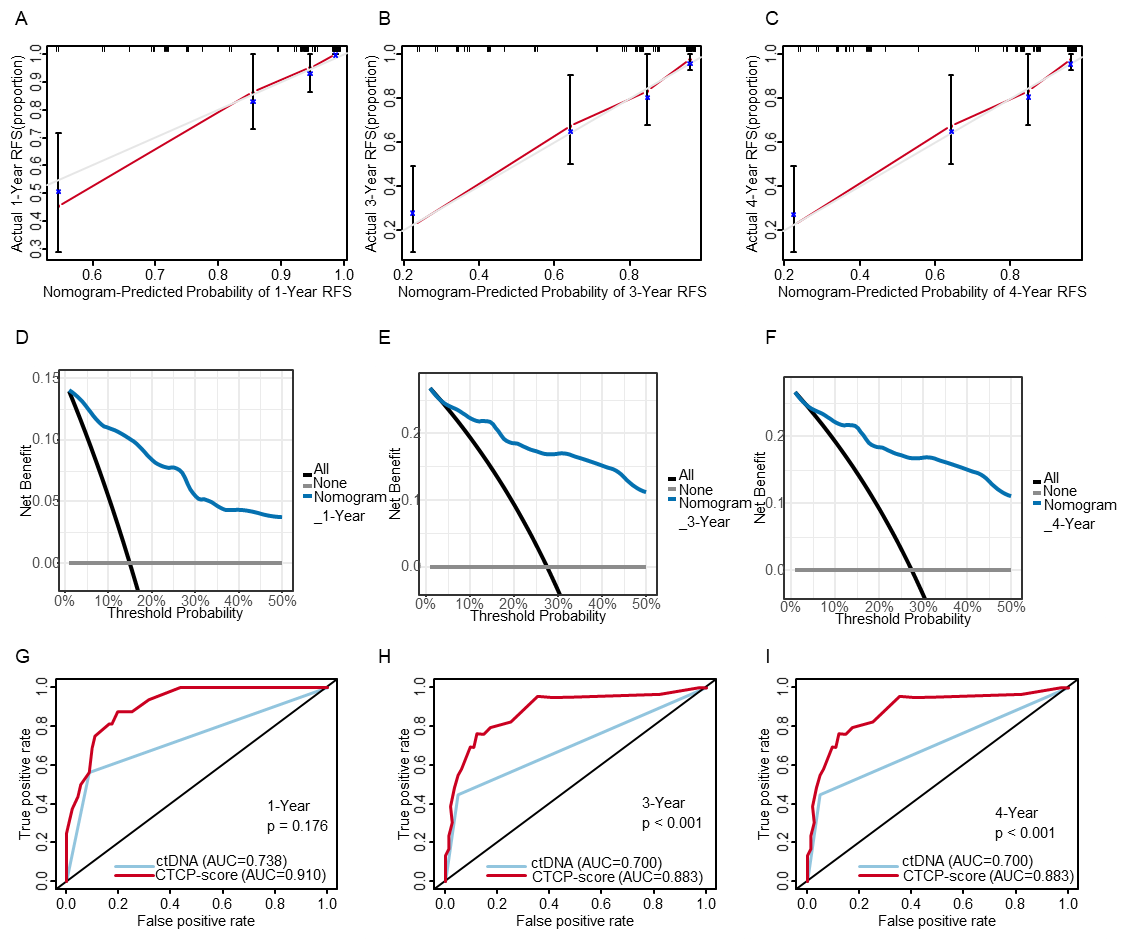


(A-C) The Calibration curves present the concordance between model prediction and actual distribution for 1-, 3- and 4-year RFS. Gray line: reference line. Red line: the prediction curve generated by the nomogram model. (D-F) The DCA curves show the net clinical benefit of the application of the CTCP model to predict 1-, 3- and 4- year RFS. (G-I) Comparison of the CTCP model with ctDNA using ROC analyses for predicting 1-, 3- and 4-year RFS.

CTCP: **ct**DNA and **c**linico**p**athological risk factors; RFS: recurrence-free survival; ctDNA: circulating tumor DNA; ROC: receiver operating characteristic; DCA: decision curve analysis.

**Figure S4** Evaluation of the CTCP model for predicting 1-year RFS in the validation cohort.


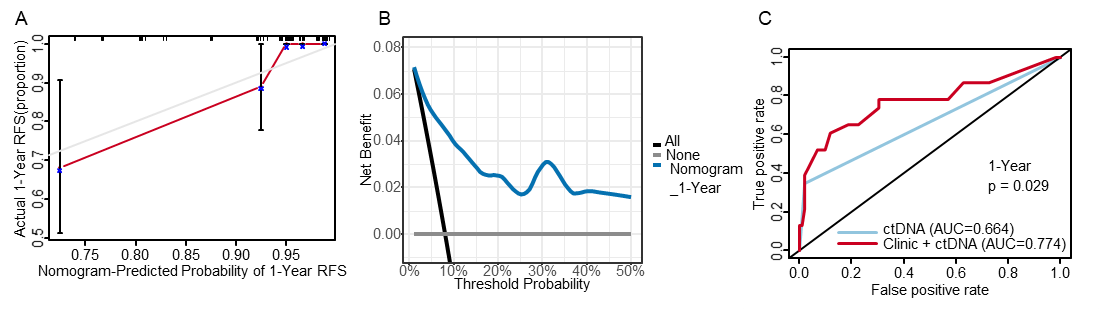


(A) The Calibration curves show the concordance between model prediction and actual distribution for 1-year RFS. Gray line: reference line. Red line: the prediction curve generated by the nomogram model. (B) The DCA curves show the net clinical benefit of the application of the CTCP model to predict 1-year RFS. (C) Comparison of the CTCP model with ctDNA using ROC analyses for predicting 1- year RFS.

CTCP: **ct**DNA and **c**linico**p**athological risk factors; RFS: recurrence-free survival; ctDNA: circulating tumor DNA; ROC: receiver operating characteristic; DCA: decision curve analysis.

**Table S1** The relationship analysis between postoperative ctDNA status and clinicopathological parameters

| **Clinicopathological parameters** |  | **ctDNA status** | | **P value** |
| --- | --- | --- | --- | --- |
|  | Overall | negative | positive |  |
|  | (N=108) | (N=91) | (N=17) |  |
| Age |  |  |  |  |
| <=60 | 50 (46.3%) | 43 (47.3%) | 7 (41.2%) | 0.844 |
| >60 | 58 (53.7%) | 48 (52.7%) | 10 (58.8%) |  |
| Sex |  |  |  |  |
| Male | 69 (63.9%) | 56 (61.5%) | 13 (76.5%) | 0.367 |
| Female | 39 (36.1%) | 35 (38.5%) | 4 (23.5%) |  |
| Tumor location |  |  |  |  |
| Left | 80 (74.1%) | 66 (72.5%) | 14 (82.4%) | 0.584 |
| Right | 28 (25.9%) | 25 (27.5%) | 3 (17.6%) |  |
| Histologic type |  |  |  |  |
| Adenocarcinoma | 98 (90.7%) | 83 (91.2%) | 15 (88.2%) | 1 |
| Mucinous | 10 (9.26%) | 8 (8.79%) | 2 (11.8%) |  |
| Histopathological differentiation grade | |  |  |  |
| Poor | 9 (8.91%) | 8 (9.30%) | 1 (6.67%) | 1 |
| Medium/Well | 92 (91.1%) | 78 (90.7%) | 14 (93.3%) |  |
| Stage |  |  |  |  |
| I | 18 (16.7%) | 18 (19.8%) | 0 (0%) | <0.001 |
| II | 44 (40.7%) | 42 (46.2%) | 2 (11.8%) |  |
| III | 46 (42.6%) | 31 (34.1%) | 15 (88.2%) |  |
| Lymphatic/vascular invasion |  |  |  |  |
| No | 87 (80.6%) | 76 (83.5%) | 11 (64.7%) | 0.143 |
| Yes | 21 (19.4%) | 15 (16.5%) | 6 (35.3%) |  |
| Nerve invasion |  |  |  |  |
| No | 91 (84.3%) | 80 (87.9%) | 11 (64.7%) | 0.04 |
| Yes | 17 (15.7%) | 11 (12.1%) | 6 (35.3%) |  |
| MSI status |  |  |  |  |
| MSS | 100 (92.6%) | 83 (91.2%) | 17 (100%) | 0.444 |
| MSI-H | 8 (7.41%) | 8 (8.79%) | 0 (0%) |  |

MSS: microsatellite stable; MSI-H: microsatellite instability-high.

**Table S2** The sensitivity and specificity of postoperative ctDNA status for predicting RFS at 6-48 months

| **Months** | **Sensitivity** | **Specificity** | **Positive predictive value** | **Negative predictive value** |
| --- | --- | --- | --- | --- |
| 6 | 60.0% | 86.4% | 17.6% | 97.8% |
| 12 | 56.3% | 91.3% | 52.9% | 92.3% |
| 18 | 52.2% | 92.7% | 63.6% | 88.8% |
| 24 | 49.6% | 94.7% | 73.4% | 86.5% |
| 30 | 51.8% | 95.9% | 79.8% | 86.5% |
| 36 | 44.7% | 93.8% | 73.0% | 81.8% |
| 42 | 44.7% | 95.5% | 78.8% | 82.1% |
| 48 | 44.7% | 92.3% | 68.7% | 81.6% |

ctDNA: circulating tumor DNA; RFS: recurrence-free survival

**Table S3** The sensitivity and specificity of the CTCP model for predicting RFS at 6-48 months

| **Months** | **Sensitivity** | **Specificity** | **Positive predictive value** | **Negative predictive value** |
| --- | --- | --- | --- | --- |
| 6 | 100.0% | 62.1% | 11.4% | 100.0% |
| 12 | 87.5% | 80.4% | 43.8% | 97.4% |
| 18 | 85.6% | 84.1% | 56.9% | 96.0% |
| 24 | 87.5% | 88.2% | 68.4% | 96.0% |
| 30 | 88.0% | 89.2% | 71.6% | 96.0% |
| 36 | 76.1% | 85.4% | 66.3% | 90.4% |
| 42 | 76.1% | 81.8% | 61.2% | 90.1% |
| 48 | 76.1% | 84.6% | 65.1% | 90.3% |

CTCP: **ct**DNA and **c**linico**p**athological risk factors; RFS: recurrence-free survival; ctDNA: circulating tumor DNA;
